# The Influence of Lead and *Acyrthosiphon pisum* (Harris) on Generation of *Pisum sativum* Defense Signaling Molecules and Expression of Genes Involved in Their Biosynthesis

**DOI:** 10.3390/ijms241310671

**Published:** 2023-06-26

**Authors:** Agnieszka Woźniak, Jacek Kęsy, Paulina Glazińska, Wojciech Glinkowski, Dorota Narożna, Jan Bocianowski, Renata Rucińska-Sobkowiak, Van Chung Mai, Włodzimierz Krzesiński, Sławomir Samardakiewicz, Beata Borowiak-Sobkowiak, Mateusz Labudda, Philippe Jeandet, Iwona Morkunas

**Affiliations:** 1 Department of Plant Physiology, Faculty of Agriculture, Horticulture and Bioengineering, Poznań University of Life Sciences, Wołyńska 35, 60-637 Poznan, Poland; agnieszkam.wozniak@gmail.com; 2 Department of Plant Physiology and Biotechnology, Faculty of Biological and Veterinary Sciences, Nicolaus Copernicus University in Toruń, Lwowska 1, 87-100 Torun, Poland; jacek.kesy@umk.pl (J.K.); paulina.glazinska@umk.pl (P.G.); w_glinkowski@o2.pl (W.G.); 3 Department of Biochemistry and Biotechnology, Faculty of Agriculture, Horticulture and Bioengineering, Poznań University of Life Sciences, Dojazd 11, 60-632 Poznan, Poland; dorota.narozna@up.poznan.pl; 4 Department of Mathematical and Statistical Methods, Faculty of Agriculture, Horticulture and Bioengineering, Poznań University of Life Sciences, Wojska Polskiego 28, 60-637 Poznan, Poland; jan.bocianowski@up.poznan.pl; 5 Department of Plant Ecophysiology, Faculty of Biology, Adam Mickiewicz University, Uniwersytetu Poznańskiego 6, 61-614 Poznan, Poland; renatar@amu.edu.pl; 6 Department of Biology and Application, Faculty of Biology, Vinh University, Le Duan 182, 43108 Vinh, Nghe An Province, Vietnam; chung.uni@gmail.com; 7 Department of Vegetable Crops, Faculty of Agriculture, Horticulture and Bioengineering, Poznań University of Life Sciences, Dąbrowskiego 159, 60-594 Poznan, Poland; wlodzimierz.krzesinski@up.poznan.pl; 8 Laboratory of Electron and Confocal Microscopy, Faculty of Biology, Adam Mickiewicz University, Uniwersytetu Poznańskiego 6, 61-614 Poznan, Poland; sas@amu.edu.pl; 9 Department of Entomology and Environmental Protection, Faculty of Agriculture, Horticulture and Bioengineering, Poznań University of Life Sciences, Dąbrowskiego 159, 60-594 Poznan, Poland; beata.borowiak@up.poznan.pl; 10 Department of Biochemistry and Microbiology, Institute of Biology, Warsaw University of Life Sciences, Nowoursynowska 159, 02-776 Warsaw, Poland; mateusz_labudda@sggw.edu.pl; 11 Research Unit “Induced Resistance and Plant Bioprotection”, RIBP USC-INRAe 1488, University of Reims, 51100 Reims, France; philippe.jeandet@univ-reims.fr

**Keywords:** lead, pea aphid, jasmonates, ethylene, genes encoding enzymes of phytohormone biosynthesis, *Pisum sativum*

## Abstract

The main aim of this study was to understand the regulation of the biosynthesis of phytohormones as signaling molecules in the defense mechanisms of pea seedlings during the application of abiotic and biotic stress factors. It was important to identify this regulation at the molecular level in *Pisum sativum* L. seedlings under the influence of various concentrations of lead—i.e., a low concentration increasing plant metabolism, causing a hormetic effect, and a high dose causing a sublethal effect—and during feeding of a phytophagous insect with a piercing-sucking mouthpart—i.e., pea aphid (*Acyrthosiphon pisum* (Harris)). The aim of the study was to determine the expression level of genes encoding enzymes of the biosynthesis of signaling molecules such as phytohormones—i.e., jasmonates (JA/MeJA), ethylene (ET) and abscisic acid (ABA). Real-time qPCR was applied to analyze the expression of genes encoding enzymes involved in the regulation of the biosynthesis of JA/MeJA (lipoxygenase 1 (*LOX1*), lipoxygenase 2 (*LOX2*), 12-oxophytodienoate reductase 1 (*OPR1*) and jasmonic acid-amido synthetase (*JAR1*)), ET (1-aminocyclopropane-1-carboxylate synthase 3 (*ACS3*)) and ABA (9-*cis*-epoxycarotenoid dioxygenase (*NCED*) and aldehyde oxidase 1 (*AO1*)). In response to the abovementioned stress factors—i.e., abiotic and biotic stressors acting independently or simultaneously—the expression of the *LOX1*, *LOX2*, *OPR1*, *JAR1*, *ACS3*, *NCED* and *AO1* genes at both sublethal and hormetic doses increased. Particularly high levels of the relative expression of the tested genes in pea seedlings growing at sublethal doses of lead and colonized by *A. pisum* compared to the control were noticeable. A hormetic dose of lead induced high expression levels of the *JAR1*, *OPR1* and *ACS3* genes, especially in leaves. Moreover, an increase in the concentration of phytohormones such as jasmonates (JA and MeJA) and aminococyclopropane-1-carboxylic acid (ACC)-ethylene (ET) precursor was observed. The results of this study indicate that the response of pea seedlings to lead and *A. pisum* aphid infestation differed greatly at both the gene expression and metabolic levels. The intensity of these defense responses depended on the organ, the metal dose and direct contact of the stress factor with the organ.

## 1. Introduction

Plants are exposed to various abiotic and biotic stress factors in their natural environment [1]. The effects of multiple stressors frequently occur simultaneously or sequentially. Therefore, as reported by Simmons et al. [2], the impacts of stress factors can combine additively or, as a result, synergistic or antagonistic effects occur. According to the deliberations of Piggott and co-authors [3], in the ecological multiple stressor context, it most commonly relates to an additive effects model. In light of the above, synergism is being defined as a cumulative effect of multiple stressors that is bigger than the additive sum of the effects triggered by the stress factors acting independently [4]. In turn, the term “antagonism” is defined as a cumulative effect that is less than additive. Moreover, it is suggested that when the two stress factors act together, they synergistically alleviate or inhibit their individual visual effects even more than under controlled conditions [3], but this is not a rule in every experimental setting.

**Figure 1:**
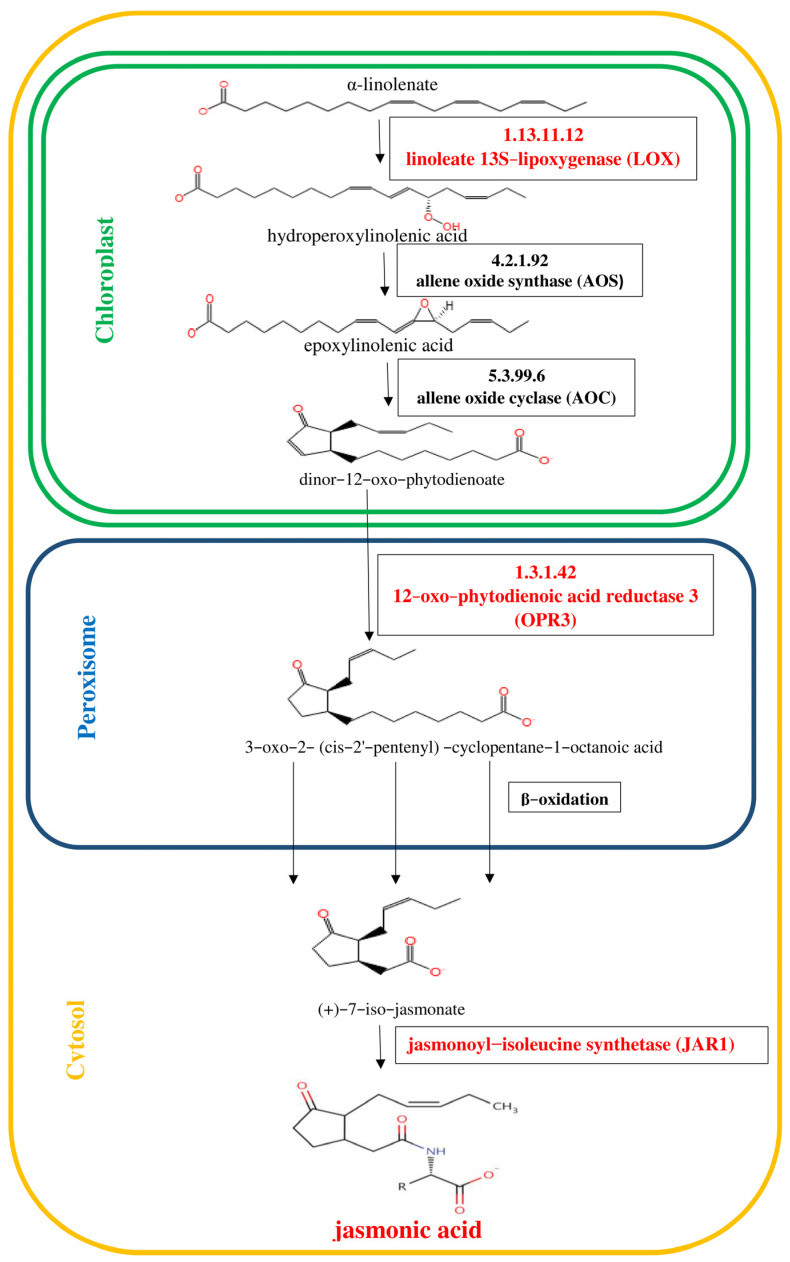
Octadecanoid pathway of jasmonic acid (JA) biosynthesis [28]. The initial steps of JA biosynthesis including lipoxygenase (LOX), allene oxide synthase (AOS) and allene oxide cyclase (AOC) lead to the production of 12-oxo-phytodienoic acid (12-OPDA). 12-OPDA is then transported to the peroxisome and reduced to 3-oxo-2-(2′(Z)-pentenyl)-cyclopentane-1-octanoic acid (OPC-8:0), which undergoes three rounds of β-oxidation to yield JA. Genes encoding enzymes involved in the biosynthesis of JA, whose expression was studied, are highlighted in red in the scheme.

**Figure 2:**
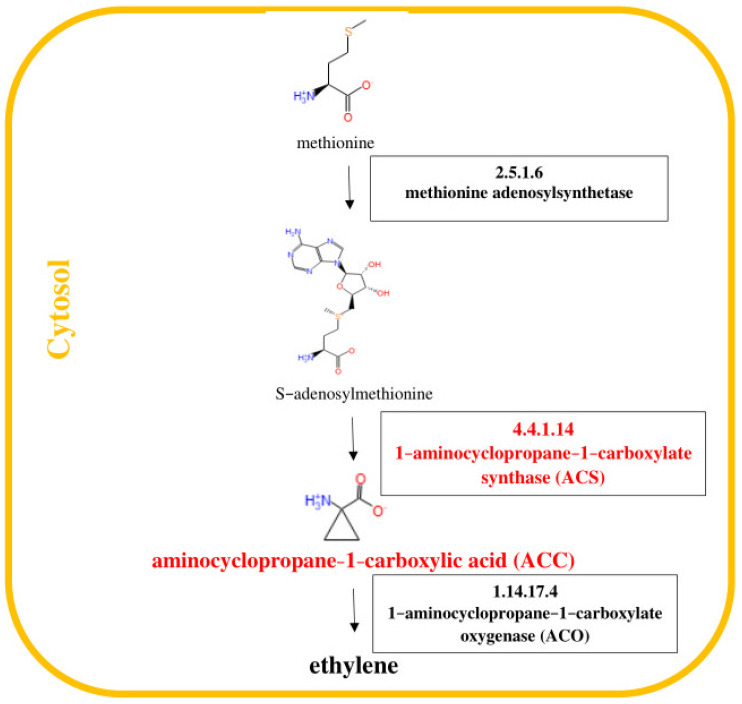
Pathway of ethylene (ET) biosynthesis [29]. Genes encoding enzymes involved in the biosynthesis of ET, whose expression was studied, are highlighted in red in the scheme.

**Figure 3:**
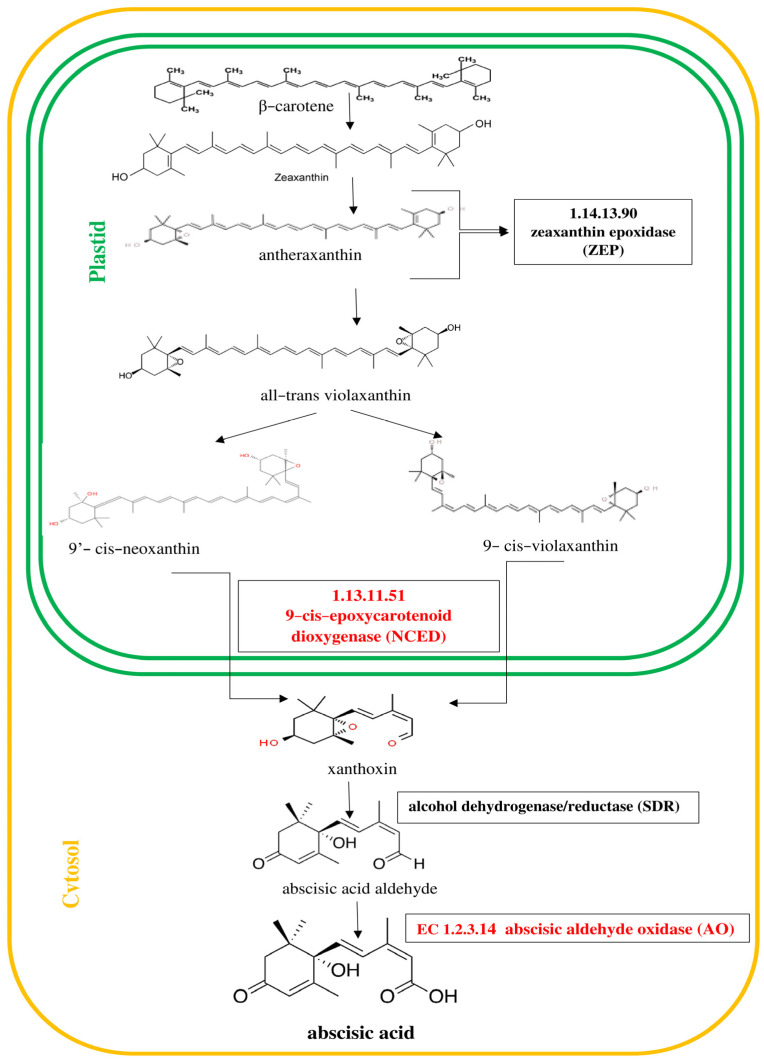
Pathway of abscisic acid (ABA) biosynthesis [30]. Genes encoding enzymes involved in the biosynthesis of ABA, whose expression was studied, are highlighted in red in the scheme.

Numerous studies have revealed that phytohormones such as jasmonic acid, ethylene and abscisic acid are components of plant defense responses [5]. The signaling pathways regulated by these molecules play key roles in the crosstalk between biotic and abiotic stress signaling [1,6,7]. It is known that jasmonates are fatty-acid-derived hormones that are structurally similar to animal prostaglandins and ubiquitous in the plant kingdom [8]. The best known compounds belonging to jasmonates are jasmonic acid (JA) and its methyl ester—methyl jasmonate (MJ) [9]. Jasmonates act not only as powerful signals activating plant defenses against herbivores, pathogens, wounding and abiotic stress but also as regulatory molecules in many developmental processes. The octadecanoid pathway of jasmonic acid (JA) biosynthesis is shown in Figure 1. In turn, ethylene is synthesized from L-methionine through the intermediates *S*-adenosyl-L-methionine (SAM) and 1-aminocyclopropane-1-carboxylate (ACC) as shown in Figure 2. Cloning and analysis of 1-aminocyclopropane-1-carboxylate synthase (ACC synthase, EC 4.4.1.14) genes from numerous plants indicated that they form a multigene family that is differentially regulated. The ABA hormone has been associated with plant tolerance to adverse environmental conditions [10,11,12]. The scheme of the biosynthesis of the sesquiterpenoid hormone abscisic acid (ABA) is displayed in Figure 3. ABA is derived from C40 epoxycarotenoid precursors through oxidative cleavage taking place in plastids [13]. Then, the C15 intermediate xanthoxin is converted to ABA by a two-step reaction via ABA-aldehyde in the cytosol.

In the present experimental work, we focus on the impact of the heavy metal lead at hormetic (0.075 mM Pb(NO_3_)_2_) and sublethal (0.5 mM Pb(NO_3_)_2_) doses and of the crosstalk between lead and a biotic stressor—i.e., pea aphid (*Acyrthosiphon pisum* (Harris)), a phytophagous insect with a piercing-sucking mouthpart—on the expression of genes encoding enzymes of the biosynthesis of phytohormones such as jasmonates (JA/MeJA), ethylene (ET) and abscisic acid (ABA). This is the first report revealing the effect of an abiotic stress factor, lead (Pb), at hormetic and sublethal doses and during the combination of Pb stress and pea aphid *A. pisum* acting as a biotic factor on the expression of genes encoding enzymes of the biosynthesis of JA/MeJA, ET and ABA. The second objective of this work is to study the effect of Pb on the concentrations of JA/MeJA and ACC-ET precursor, and then to determine cross-interactions of both stresses. The same experimental model, including the application of Pb at hormetic and sublethal doses and the combination of Pb and *A. pisum*, was applied in our previous research [6,14]. In the referred study, the growth of pea seedlings in the context of the above Pb doses, selected changes in the metabolome and the effect of lead on behavioral responses of *A. pisum* during probing in plant tissues were shown. The present study is a continuation of our experimental work performed on the same model of *Pisum sativum* L.cv. Cysterski–*A. pisum* in order to investigate the defense mechanisms of pea against hormetic and sublethal Pb doses and during the cross-talk between Pb and pea aphids. It should be emphasized that the phenomenon of hormesis is extremely interesting in certain research on the responses of plants to insects at hormetic doses of heavy metals. The toxic effects of heavy metals on plants are generally well documented in the literature [15,16,17,17,18,19,20,21].

Hormesis was described by Calabrese and Agathokleous [22] as a fundamental evolutionary adaptive strategy. Moreover, the above authors reported that hormesis has emerged as a fundamental dose–response phenomenon occurring from local to global scales [23,24]. Additionally, questions have also been raised regarding the ecological and evolutionary consequences that hormesis may have [25,26,27]. Furthermore, our previous review paper discussed the impact of heavy metals on the growth of plants at different concentrations, paying particular attention to the hormesis effect [18]. Study of the hormesis phenomenon has been considered not only in the framework of plant growth stimulation but also as an adaptive response of plants to a low level of stress, which in turn can play an important role in their responses to biotic stressors.

## 2. Results

### 2.1. The Effect of Lead and A. pisum on Jasmonates Accumulation in Pea Seedlings

**Figure 4:**
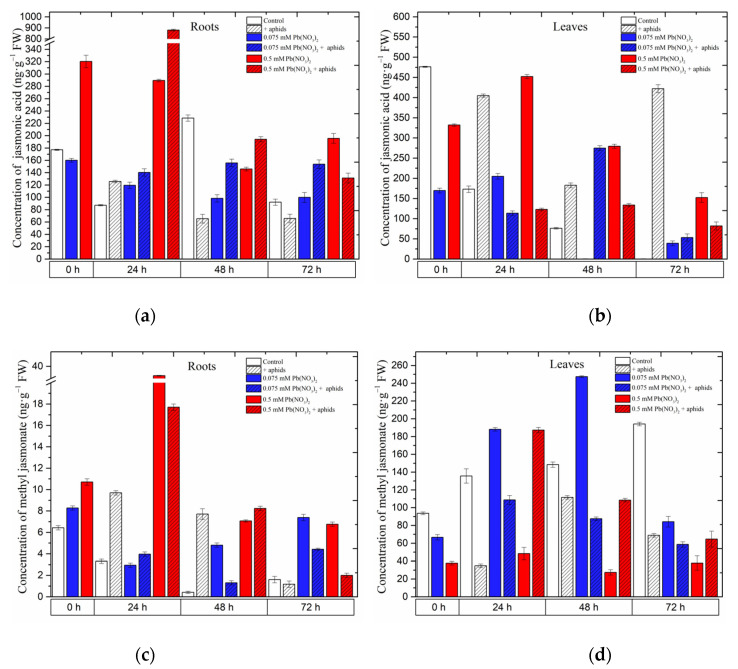
Effect of lead and *A. pisum* on accumulation of jasmonic acid (JA) (**a**,**b**) and methyl jasmonate (MeJA) (**c**,**d**) in the organs of pea seedlings. The data were obtained in three independent experiments and were statistically analyzed using ANOVA (*p* < 0.001).

Already at the beginning of the experiment, i.e., 4 days after the administration of lead and before aphid transfer to pea seedlings, a significant accumulation of jasmonates, namely jasmonic acid (JA) and methyl jasmonate (MeJA), was recorded (Figure 4) in the roots of pea seedlings growing on the Hoagland medium with 0.075 and 0.5 mM Pb(NO_3_)_2_. The concentrations of JA and MeJA in the roots of pea seedlings, particularly those growing with the sublethal dose of Pb, were more than 1.5 times higher than in the control. In turn, the concentration of MeJA in the roots of pea seedlings growing at the hormetic (0.075 mM Pb(NO_3_)_2_) dose was 1.3 times higher than in the control. Statistical analysis confirmed the significance of the differences in these results (Table 1). Moreover, the highest JA level was recorded in 24-h roots (900 ng g^−1^ FW) of pea seedlings growing at the sublethal (0.5 mM Pb(NO_3_)_2_) dose and colonized by pea aphids *A. pisum*. The content of JA was higher than that in the roots of the seedlings treated only with the sublethal dose of Pb and not infested with aphids and other experimental variants. At the subsequent time point, i.e., at 48 h, higher levels of MeJA were recorded in the roots of pea seedlings infested by aphids and exposed to hormetic (0.075 mM Pb(NO_3_)_2_) and sublethal (0.5 mM Pb(NO_3_)_2_) doses and during the cross-talk interaction compared to the control. In turn, in 24-h and 48-h leaves of pea seedlings growing on the medium with the hormetic (0.075 mM Pb(NO_3_)_2_) dose, a significant increase in MeJA in relation to the control was noted. Additionally, the concentration of MeJA in the 0.5 mM Pb(NO_3_)_2_ + aphids variant was higher than that in the control and comparable to the 0.075 mM Pb(NO_3_)_2_) variant.

### 2.2. The Effect of Lead and A. pisum on 1-Aminocyclopropane-1-carboxylate (ACC)—Ethylene Precursor Accumulation in Pea Seedlings

**Figure 5:**
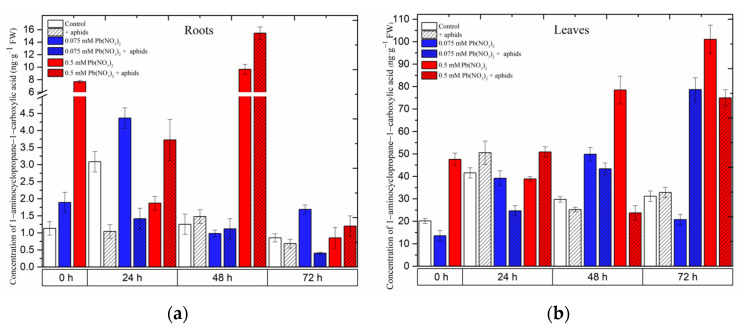
Effect of lead and *A. pisum* on the accumulation of 1-aminocyclopropane-1-carboxylate (ACC) (**a**,**b**) in the organs of pea seedlings. The data were obtained from three independent experiments and were statistically analyzed using ANOVA (*p* < 0.001).

Hormetic (0.075 mM Pb(NO_3_)_2_) and sublethal (0.5 mM Pb(NO_3_)_2_) doses and cross-talk of lead and pea aphid interaction caused accumulation of 1-aminocyclopropane-1-carboxylate (ACC) in the organs of pea seedlings (Figure 5). A significant accumulation was recorded for ACC in the roots and leaves of pea seedlings growing on the Hoagland medium, especially with the sublethal dose (i.e., 0.5 mM Pb(NO_3_)_2_) variant or/and the 0.5 mM Pb(NO_3_)_2_ + aphids variant. The hormetic dose (solution of 0.075 mM Pb(NO_3_)_2_) induced ACC accumulation in the roots of 24-h pea seedlings after treatment, and then ACC concentrations were reduced to lower levels; however, the solution of 0.5 mM Pb(NO_3_)_2_ enhanced the ACC content, peaking at 48 h. The combined effect of lead and aphid infestation (0.5 mM Pb(NO_3_)_2_ + aphids variant) caused an increase in ACC levels from 24 h to 48 h, then declined at 72 h after treatment (Figure 5a).

Meanwhile, in the leaves of pea seedlings, the ACC content increased continuously along the experiment: at the dose of 0.5 mM Pb(NO_3_)_2_, the highest recorded level of ACC was 101.0709 ng.g^−1^ FW at 72 h, which is 3.24-fold higher than in the control. This changing trend in ACC levels was also noted in the combination between aphid impact and 0.075 mM Pb(NO_3_)_2_: the highest level of ACC in this case was 75.0047 ng.g^−1^ FW at 72 h, which is 2.40-fold higher than in the control (Figure 5b). ANOVA results showed that the differences in the ACC contents in lead- and aphid-treated leaves and the control plants were highly statistically significant at 72 h. However, no statistical difference was recorded between ACC levels in variants of aphid infestation and the control at that time point.

### 2.3. The Effect of Lead and A. pisum on Expression Levels of Lipoxygenase (LOX1)

Remarkable differences in the expression of *Lipoxygenase 1* (*LOX1*), a gene encoding lipoxygenase, an important enzyme in the JA biosynthesis pathway, were observed during the experiments.

**Figure 6:**
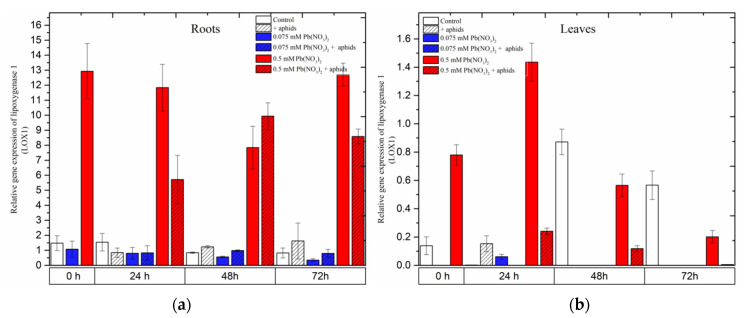
Expression of *lipoxygenase (LOX1)* in roots (**a**) and leaves (**b**) of pea seedlings exposed to lead and *A. pisum*. Gene expression level was assessed by RT-qPCR. *Phosphoprotein phosphatase 2A* (*PP2A*) was used as the reference gene. The data were obtained from three independent experiments and statistically analyzed using ANOVA (*p* < 0.001).

The hormetic (0.075 mM Pb(NO_3_)_2_) dose and cross-talk of 0.075 mM Pb(NO_3_)_2_ and aphids did not cause any increase in *LOX1* gene expression. The expression of *LOX1* in the roots of seedlings remained at a low level in the control and all the treated variants during the experiments, while there was a lack of expression of this gene in the leaves of pea seedlings at almost all time points (Figure 6). Furthermore, it should be emphasized that the sublethal (0.5 mM Pb(NO_3_)_2_) dose considerably induced the expression of *LOX1* in the roots of the seedlings, and more strongly than that in the leaves. A very high level of *LOX1* transcripts in roots from 0.5 mM Pb(NO_3_)_2_ variants was maintained at all time points, i.e., from the starting of the experiments to 72 h. Additionally, the expression of *LOX1* in roots from the 0.5 mM Pb(NO_3_)_2_ + aphids variants was also high, and significantly higher than in the control and other variants, but lower than in roots from the 0.5 mM Pb(NO_3_)_2_ variants (24 h and 72 h). The statistical analysis confirmed the significance of the differences in these results.

### 2.4. The Effect of Lead and A. pisum on Expression Levels of Lipoxygenase 2 (LOX2)

**Figure 7:**
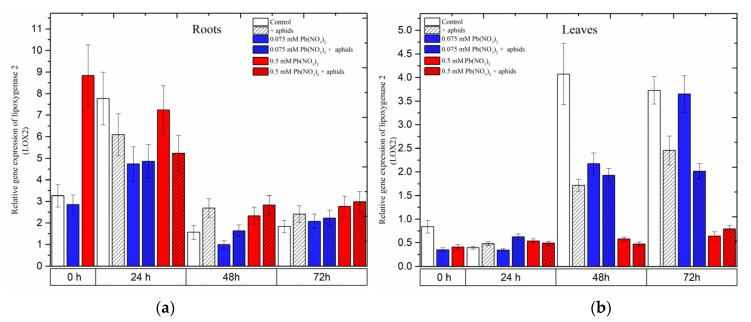
Expression of *lipoxygenase* (*LOX2*) in roots (**a**) and leaves (**b**) of pea seedlings exposed to lead and *A. pisum*. Gene expression level was assessed by RT-qPCR. *Phosphoprotein phosphatase 2A* (*PP2A*) was used as a reference gene. The data were obtained from three independent experiments and statistically analyzed using ANOVA (*p* < 0.001).

Already at the beginning of the experiment, i.e., 4 days after the administration of lead and before aphid transfer to pea seedlings, a significant increase in the expression level of *LOX2* was noted; it was 2.5 times higher than in the control and 0.075 mM Pb(NO_3_)_2_ variant (Figure 7). Then, at 24 h, exposure of the pea seedlings to stress factors was associated with downregulation of the *LOX2* gene in the roots *(*Figure 7a). At the next time points (48 h), the expression of *LOX2* decreased. However, it remained higher in the roots of pea seedlings infested by aphids (+aphids variant) and the roots of pea seedlings of the 0.5 mM Pb(NO_3_)_2_ variant and the 0.5 mM Pb^2+^ + aphids variant than in the control.

In turn, at the beginning of the experiment, the level of *LOX2* transcripts was two times lower in pea seedling leaves from hormetic and sublethal variants than in the control. At later time points, the expression levels of *LOX2* in pea seedling leaves increased continuously as a function of time, both in the control and the hormetic variants (0.075 mM Pb(NO_3_)_2_ variant and 0.075 mM Pb^2+^ + aphids). Furthermore, at 48 h, downregulation of the *LOX2* gene was observed in the leaves of pea seedlings from all stress variants compared to the control. A similar trend was observed in 72-h leaves, with the exception of 72-h leaves from the 0.075 mM Pb(NO_3_)_2_ variant.

### 2.5. The Effect of Lead and A. pisum on Expression Levels of 12-Oxophytodienoate Reductase 1 (OPR1)

**Figure 8:**
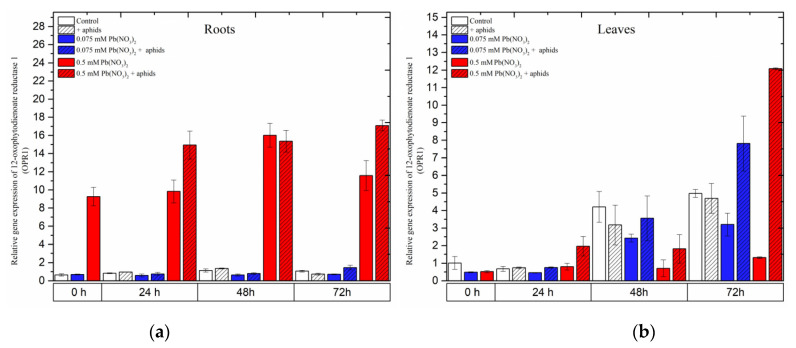
Expression of *12-oxophytodienoate reductase* 1 (*OPR1*) in roots (**a**) and leaves (**b**) of pea seedlings exposed to lead and *A. pisum*. Gene expression level was assessed by RT-qPCR. *Phosphoprotein phosphatase 2A* (*PP2A*) was used as the reference gene. The data were obtained from three independent experiments and statistically analyzed using ANOVA (*p* < 0.001).

A remarkable difference in the expression of the *12-oxophytodienoate reductases* (*OPR1*) gene, the gene encoding 12-oxophytodienoate reductase 1, another important enzyme in the JA biosynthesis pathway, was recorded in the organs of the pea seedlings. Treatment of pea seedling roots with the hormetic dose of 0.075 mM Pb(NO_3_)_2_ or cross-talk between the hormetic dose of 0.075 mM Pb(NO_3_)_2_ and aphids did not induce *OPR1* gene expression. *OPR1* expression in the roots of the seedlings remained at low levels in the control variant and all treated variants during the experimental times (Figure 8a). Meanwhile, treating pea seedlings with the sublethal dose, i.e., 0.5 mM Pb(NO_3_)_2_ or 0.5 mM Pb(NO_3_)_2_ and aphids, strongly induced expression of the *OPR1* gene during the experiments. The highest expression level of *OPR1* was recorded at 48 h as a result of treatment with 0.5 mM Pb(NO_3_)_2_ and at 72 h during 0.5 mM Pb(NO_3_)_2_ and aphid combination. ANOVA results showed that the differences in the expression levels of the *OPR1* gene in pea seedling roots from the 0.5 mM Pb(NO_3_)_2_ and 0.5 mM Pb(NO_3_)_2_ + aphids variants and the control were highly statistically significant at all time points.

In leaves, the expression levels of *OPR1* in all variants seemed to continuously increase from the start of the experiments until 72 h. The combined effect of the sublethal dose of lead and aphids caused the highest expression levels of the *OPR1* gene at 72 h after treatment. In parallel, an upregulation of *OPR1* in pea seedling leaves from the hormetic (0.075 mM Pb(NO_3_)_2_) dose and infestation by aphids was noted. The expression level of *OPR1* in the above variant was 1.5 times higher than in the control. At that time point, ANOVA recorded differences in the expression levels of *OPR1* in the 0.075 mM Pb(NO_3_)_2_ and aphid and 0.5 mM Pb(NO_3_)_2_+ aphids variants and the control, which were statistically significant.

### 2.6. The Effect of Lead and A. pisum on Expression Levels of Jasmonic Acid-Amido Synthetase (JAR1)

In the organs of pea seedlings, the expression levels of *JAR1* in most of the variants seemed to continuously increase from the beginning of the experiments until 48 h and then declined at 72 h.

**Figure 9:**
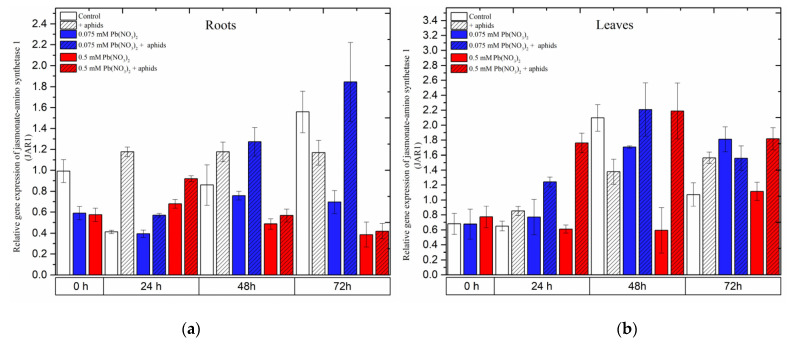
Expression of *jasmonic acid-amido synthetase (JAR1)* in roots (**a**) and leaves (**b**) of pea seedlings exposed to lead and *A. pisum*. Gene expression level was assessed by RT-qPCR. *Phosphoprotein phosphatase 2A* (*PP2A*) was used as the reference gene. The data were obtained from three independent experiments and statistically analyzed using ANOVA (*p* < 0.001).

In the roots, aphid infestation induced *JAR1* expression 24 h after treatment, which remained at high levels at all experimental time points. The combined effect of aphid and a low lead concentration (in 0.075 mM Pb(NO_3_)_2_ solution) yielded a continuous increase in *JAR1* expression from 24 to 72 h, with relative gene expression increasing from 0.57 to 1.85. However, only a high concentration of lead (0.5 mM Pb(NO_3_)_2_ solution) displayed an effect without and with aphid at 24 h after treatment; in those cases, the relative expression of *JAR1* was higher than that in the control (Figure 9a).

In the leaves of pea seedlings, lead (at both doses) and aphids induced differential expression of *JAR1* in most of the experimental variants. Regarding the individual effect of aphids or lead, the expression of *JAR1* increased over the course of the experiment and reached the highest level at 72 h, with relative gene expressions of 1.6 under aphid infestation, 1.8 with the 0.075 mM Pb(NO_3_)_2_ solution and 1.1 with the 0.5 mM Pb(NO_3_)_2_ solution. However, the combined effect of aphids with the two lead concentrations enhanced *JAR1* expression, strongly increasing from 24 h to 48 h and then slightly declining at 72 h. The highest levels of *JAR1* expression at 48 h were 2.2 following aphid infestation with the 0.075 mM Pb(NO_3_)_2_ solution and 2.2 following aphid infestation with the 0.5 mM Pb(NO_3_)_2_ solution (Figure 9a). The ANOVA recorded differences in the expression levels of *JAR1* in most of the treated variants and the control, which were highly statistically significant at all tested time points.

### 2.7. The Effect of Lead and A. pisum on Expression Levels of 1-Aminocyclopropane-1-carboxylate Synthase 3 (ACS3)

**Figure 10:**
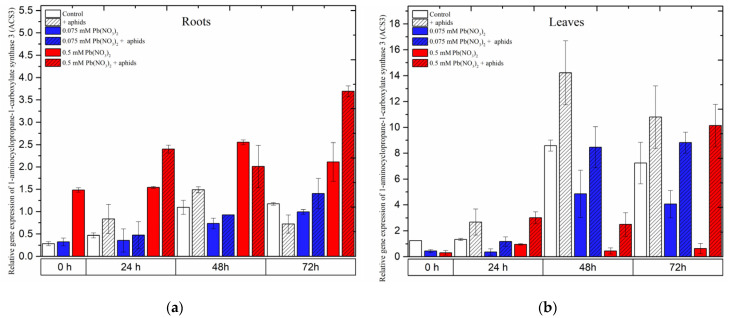
Expression of *1-aminocyclopropane-1-carboxylate synthase 3* (*ACS3*) in roots (**a**) and leaves (**b**) of pea seedlings exposed to lead and *A. pisum*. Gene expression level was assessed by RT-qPCR. *Phosphoprotein phosphatase 2A* (*PP2A*) was used as the reference gene. The data were obtained from three independent experiments and statistically analyzed using ANOVA (*p* < 0.001).

Expression of the *1-aminocyclopropane-1-carboxylate synthase 3 (ACS3)* gene was strongly induced in pea seedling roots growing in the medium with a high concentration of lead, i.e., 0.5 mM Pb(NO_3_)_2_. Following treatment with a sublethal dose of lead, *ACS3* demonstrated a remarkably high expression, increasing from the beginning of the experiment until 48 h and slightly declining at 72 h. However, its expression always remained higher than that in the control at all time points. Moreover, the combined effect of lead (0.5 mM Pb(NO_3_)_2_ solution) and aphids induced a higher expression of *ACS3*. The effect of these two factors caused an increase in the relative expression of *ACS3* to the highest level at 72 h after treatment (Figure 10a).

Contrary to the expression of *ACS1* in pea seedling roots, the expression of this gene in leaves was enhanced more strongly by aphids than by other stressors. Pea aphid induced rapid relative expression of *ACS3*, reaching the highest levels at 48 h and then slightly declining at 72 h.

When treating pea seedlings with both aphids and lead, *ACS3* was upregulated from the beginning of the experiment until 72 h, but its expression was always lower than in leaves infested by aphids only (Figure 10b). In 72-h leaves of pea seedlings from the 0.075 mM Pb(NO_3_)_2_ +aphids variant, the relative expression of *ACS3* was about 0.82 times higher than in the control. ANOVA results recorded differences in the expression levels of *ACS3* in variants treated by aphids without/with lead and the control, and these differences were statistically significant at all tested time points.

### 2.8. The Effect of Lead and A. pisum on Expression Levels of 9-cis-Epoxycarotenoid Dioxygenase (NCED)

The gene encoding 9-*cis*-epoxycarotenoid dioxygenase 2 (*NCED2*), an important enzyme in the biosynthesis of abscisic acid (ABA), showed different expression levels in the roots and leaves of pea seedlings as a response to different stress factors, including lead, aphids and both lead and aphids combined.

**Figure 11:**
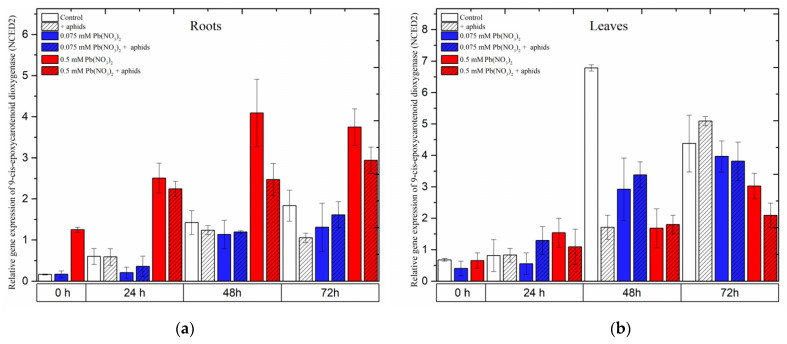
The effect of lead and *A. pisum* on expression levels of 9-*cis*-epoxycarotenoid dioxygenase (NCED) in the organs of pea seedlings. (**a**) roots, (**b**) leaves. The data were obtained from three independent experiments and statistically analyzed using ANOVA (*p* < 0.001).

In roots, it was noted that a high concentration of lead (sublethal dose of 0.5 mM Pb(NO_3_)_2_) strongly induced the expression of *NCED2*. Under the sole effect of lead, *NCED2* expression was remarkably induced after treatment with a sublethal dose, increasing from the starting of the experiment until 48 h and slightly reducing at 72 h; however, its expression was always higher than that of the control at all time points. Furthermore, the combined effect of lead (0.5 mM Pb(NO_3_)_2_ solution) and aphids induced relatively high expression levels of *NCED2*, increasing continuously from the beginning of the experiment until 72 h after treatment. The expression of *NCED2* in roots from the 0.5 mM Pb(NO_3_)_2_ + aphids variant was lower than that in the 0.5 mM Pb(NO_3_)_2_ variant. ANOVA results recorded differences in the expression levels of *NCED2* in variants treated with the sublethal dose of lead (0.5 mM Pb(NO_3_)_2_ variants) without aphids/with aphids (0.5 mM Pb(NO_3_)_2_ +aphids variant) and the control, which were highly statistically significant at all tested time points. However, a low concentration of lead (hormetic 0.075 mM Pb(NO_3_)_2_ dose) without and/or with aphid infestation (0.075 mM Pb(NO_3_)_2_ + aphids) downregulated *NCED2* gene expression when compared with the control group; the expression of this gene in the treated variants was not significantly different from that in control (Figure 11a).

Contrary to the expression of *NCED2* in the roots of the pea seedlings, this gene was differently expressed in leaves affected by lead and aphids. The relative expression of *NCED2* in all treated variants continuously increased from the beginning of the experiment until 72 h after treatment; however, the expression trends in the levels of the *NCED2* gene were different among the variants. At 24 h after treatment, *NCED2* was upregulated by the sublethal dose of lead (0.5 mM Pb(NO_3_)_2_) and the combined effect of lead and aphids (at both concentrations of 0.075 mM Pb(NO_3_)_2_ and 0.5 mM Pb(NO_3_)_2_); its expression was higher than that in the control. However, the relative expression of *NCED2* in most of the treated variants (except for aphid infestation at 72 h) was always lower than that in the control (Figure 11b).

### 2.9. The Effect of Lead and A. pisum on Expression Levels of Aldehyde Oxidase 1 (AO1)

**Figure 12:**
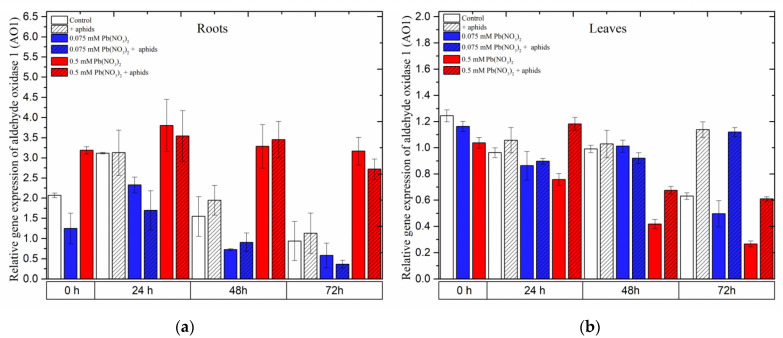
Expression of *aldehyde oxidase 1 (AO1)* in roots (**a**) and leaves (**b**) of pea seedlings exposed to lead and *A. pisum*. Gene expression level was assessed by RT-qPCR. *Phosphoprotein phosphatase 2A* (*PP2A*) was used as the reference gene. The data were obtained from three independent experiments and statistically analyzed using ANOVA (*p* < 0.001).

In the roots of pea seedlings, a high concentration of lead (0.5 mM Pb(NO_3_)_2_ solution) strongly induced the expression of the *AO1* gene, which encodes aldehyde oxidase 1 (AO1), an important enzyme in the biosynthesis of ABA. Under the sole effect of lead, *AO1* was remarkably upregulated by the sublethal dose of lead (0.5 mM Pb(NO_3_)_2_, rapidly increasing to high levels at 24 h and slightly declining at 48–72 h; however, its expression was always higher than that in the control at all time points. Similar to this evolution trend, the relative expression of *AO1* in roots of the pea seedlings was noted as a result of the combined effect of lead and aphids (0.5 mM Pb(NO_3_)_2_ + aphids variants). ANOVA results did not record any differences in the expression levels of *AO1* in variants of pea seedling roots treated with lead (in 0.5 mM Pb(NO_3_)_2_ solution) without/with aphids; however, the different expression levels of *AO1* in those variants were highly statistically significant compared to the control at all tested time points (Figure 12a).

In contrast, the expression of *AO1* in the leaves of pea seedlings was mainly upregulated by aphids. The expression levels of *AO1* in the aphid-infested variants were higher than those in the control at all time points. Increased expression of the *AO1* gene was also observed in pea seedling leaves under the effect of aphids, upon application of a sublethal dose of lead with aphid interactions (0.5 mM Pb(NO_3_)_2_ + aphids variant) at 24 h and under the effect of aphids alone or the hormetic dose and aphids (0.075 mM Pb(NO_3_)_2_ + aphids variant) at 72 h. The relative expression of *AO1* in the other experimental variants showed lower levels or no significant differences compared to the control (Figure 12b).

### 2.10. Correlation Analysis

The MANOVA results obtained for roots indicated that time (*F*_20;54_ = 308.45), variants (*F*_50;127_ = 207.93) and time × variant interactions (*F*_100;205_ = 45.88) were statistically significantly different (*p* < 0.0001) for all the ten traits jointly. The analysis of variance indicated that the main effect of time was significant for all the traits of this study, except for *LOX1* (Table 1). The main effects of the variants were significant for all observed traits; however, the time × variant interaction was statistically significant for all the traits of this study except for *NCED2* (Table 2).

The MANOVA results obtained for leaves indicated that time (*F*_20;54_ = 392.59), variants (*F*_50;127_ = 306.45) and time × variant interaction (*F*_100;205_ = 74.54) were statistically significantly different (*p* < 0.0001) for all the ten traits jointly. The analysis of variance indicated that the main effects of time, variant and time × variant interactions were significant for all the traits of this study (Table 3).

**Figure 13:**
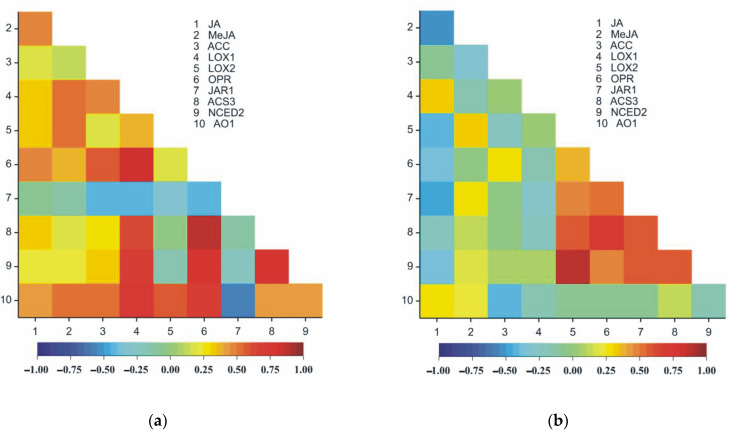
Heatmaps for Pearson’s correlation coefficients between all pairs of the ten observed traits in roots (**a**) and in leaves (**b**). The correlation coefficients ranged from −1 (blue) to 1 (red); *r*_cr;0.05_ = 0.19, *r*_cr;0.01_ = 0.25, *r*_cr;0.001_ = 0.32.

In both organs of pea seedlings (roots and leaves), significant positive correlations were observed between the following pairs of traits: JA and *LOX1*, JA and *AO1*, MeJA and *LOX2*, ACC and *OPR*, *OPR* and *ACS3*, *OPR* and *NCED2* and *ACS3* and *NCED2* (Table 3, Figure 13a,b). *LOX1* and *JAR1* were negatively correlated in both organs of the plants (roots and leaves) (Table 3, Figure 13a,b). Different signs of correlation, particularly the organs of the plant, were observed between JA and MeJA, JA and *LOX2*, JA and *OPR*, ACC and *AO1* and *OPR* and *JAR1*, as well as *LOX2* and *JAR1* (Table 3, Figure 13a,b). Eight pairs of traits were strongly correlated only in leaves (Table 3, Figure 13b); however, 18 pairs in roots were recorded.

**Table 1:** Mean squares from two-way analysis of variance of observed traits in roots.

Source of Variation	Time	Variant	Time × Variant	Residual
d.f.	2	5	10	36
JA	123694.6 ***	124364.5 ***	86281.6 ***	110.7
MeJA	361.43067 ***	249.64062 ***	142.99084 ***	0.04462
ACC	158.83 ***	78.44 **	73.7 ***	16.06
LOX1	1.7468	181.2277 ***	6.9638 ***	0.6505
LOX2	85.9859 ***	3.3316 ***	2.2319 ***	0.414
OPR	6.994 ***	432.4829 ***	5.6986 ***	0.5495
JAR1	0.50587 ***	0.85413 ***	0.44485 ***	0.01713
ACS3	1.95411 ***	5.95314 ***	0.5122 ***	0.06826
NCED2	5.0463 ***	10.1484 ***	0.2751	0.1322
AO1	9.3702 ***	8.9024 ***	0.4607 **	0.132

** *p* < 0.01; *** *p* < 0.001.

**Table 2:** Mean squares from two-way analysis of variance of observed traits in leaves.

Source of Variation	Time	Variant	Time × Variant	Residual
d.f.	2	5	10	36
JA	69066.68 ***	115201.53 ***	30854.1 ***	64.41
MeJA	7195.15 ***	24832.73 ***	6804.98 ***	18.82
ACC	2563 **	1343.7 **	1210.7 **	358.4
LOX1	0.188245 ***	0.847116 ***	0.348558 ***	0.002717
LOX2	14.71031 ***	6.22456 ***	2.02012 ***	0.04167
OPR	106.381 ***	21.2632 ***	13.8401 ***	0.4203
JAR1	2.6038 ***	1.44302 ***	0.35843 ***	0.03543
ACS3	162.62 ***	75.248 ***	24.415 ***	1.453
NCED2	35.6774 ***	5.4425 ***	4.8647 ***	0.2131
AO1	0.265663 ***	0.373335 ***	0.117172 ***	0.003367

** *p* < 0.01; *** *p* < 0.001.

**Table 3:** The correlation matrix for the traits observed in roots (above diagonal) and leaves (below).

	JA	MeJA	ACC	LOX1	LOX2	OPR	JAR1	ACS3	NCED2	AO1
JA	1	0.49 ***	0.18	0.34 **	0.31 *	0.48 ***	−0.06	0.34 **	0.21	0.42 ***
MeJA	−0.53 ***	1	0.12	0.51 ***	0.5 ***	0.36 **	−0.14	0.18	0.23	0.54 ***
ACC	−0.1	−0.31	1	0.49 ***	0.18	0.57 ***	−0.41 ***	0.28 *	0.32 *	0.52 ***
LOX1	0.31 *	−0.17	0.04	1	0.39 **	0.82 ***	−0.44 ***	0.65 ***	0.66 ***	0.71 ***
LOX2	−0.42 ***	0.32 *	−0.24	0.04	1	0.16	−0.31 *	−0.03	−0.18	0.59 ***
OPR	−0.39 **	−0.02	0.29 *	−0.2	0.39 **	1	−0.41 ***	0.87 ***	0.79 ***	0.67 ***
JAR1	−0.48 ***	0.26 *	−0.02	−0.26 *	0.48 ***	0.54 ***	1	−0.15	−0.21	−0.55 ***
ACS3	−0.23	0.12	−0.03	−0.22	0.58 ***	0.72 ***	0.58 ***	1	0.78 ***	0.40 ***
NCED2	−0.36 **	0.15	0.05	0.09	0.85 ***	0.49 ***	0.59 ***	0.57 ***	1	0.43 ***
AO1	0.27 *	0.21	−0.43 ***	−0.17	−0.06	−0.1	−0.05	0.14	−0.16	1

* *p* < 0.05; ** *p* < 0.01; *** *p* < 0.001.

## 3. Discussion

The results obtained in this work showed, for the first time, the involvement of the biosynthesis of defense phytohormones at a molecular level in edible pea (*Pisum sativum* L. Cysterski) upon exposure of the plant to varying concentrations of lead—with the low concentration potentially leading to the hormesis effect and the high concentration causing a sublethal effect—as well as during infestation by phytophagous pea aphids (*Acyrthosiphon pisum*) (Harris). These studies are valuable for explaining the defense mechanisms of pea seedlings under controlled conditions as a response to numerous stressors. First, a significant induction of the expression of genes encoding enzymes for the biosynthesis of defense phytohormones such as JA/MeJA (*LOX1*, *LOX2*, *OPR1* and *JAR1*), ET (*ACS3*) and ABA (*NCED* and *AO1*) has been demonstrated. It should be emphasized that the highest expression levels of genes encoding these enzymes, such as *LOX1*, *LOX2*, *OPR1*, *ACS3*, *NCED2* and *AO1*, were observed in roots with a sublethal dose of lead. Additionally, pea aphid infestation on pea seedlings caused a strong upregulation of the abovementioned genes in the roots growing at a sublethal lead dose at some experimental time points. This result was clearly visible for genes such as *LOX1, OPR1* and *ACS3*. Moreover, a very strong upregulation of the *JAR1* gene in roots due to the effect of aphid feeding alone or the lead hormesis dose combined with aphid infestation was reported. This result clearly proves the upregulation of the *JAR1* gene and of those mentioned above as a result of signal transmission from the leaves of pea seedlings to the roots in response to *A. pisum* infestation. Moreover, a strong upregulation of *OPR1* and *ACS3* gene expression in the leaves of pea seedlings under the influence of aphids and the cross-talk of lead and aphids was noted. It should be mentioned that changes in the expression of the analyzed genes in the roots and the leaves sometimes showed different trends, which was certainly dependent on the contact of the stressor with the pea seedling organs, its intensity and the duration. The results obtained in this work also revealed a significant increase in JA/MeJA and ET concentrations in response to abiotic (lead at sublethal and hormetic doses) and biotic (pea aphid *A. pisum* infestation) stressors acting independently or simultaneously.

Signaling networks play an important role in plant adaptation to environmental conditions and in plant survival [31]. MeJA is known to be one of the most crucial plant signaling molecules that mediates both local and systemic responses against a wide spectrum of environmental stress factors, including insect attack [32]. Jasmonates may function as fundamental phytohormones protecting plants from heavy metals and metalloids, as evidenced by the evolutionary conservation and diversity of these gene families in a large number of species of the major green plant lineages [33]. Additionally, jasmonic acid applications are effective against aphids [34]. For instance, exogenous applications of JA decreased aphid reproduction in cucumber leaves and increased the gene expression level of *OPR11* [35].

Plants exposed to abiotic stresses (heat and drought combined) and biotic stresses (grain aphid infestation) have been shown to have elevated levels of ABA. Besides the increase in ABA, grain aphid infestation further increased the jasmonic acid- and salicylic acid-dependent defenses under combined abiotic stress conditions.

In the present work, we showed the effects of lead and cross-talk of Pb and the pea aphid *A. pisum* on the expression levels of the 9-*cis*-epoxycarotenoid dioxygenase (*NCED*) and *aldehyde oxidase 1* (*AO1*) genes. The results of our current research have revealed a significant upregulation of these genes which encode important enzymes of ABA biosynthesis in the roots of pea seedlings. The concentration of ABA in this model system has been determined in previous work [6]. A significant accumulation of ABA and total salicylic acid (TSA) was recorded in the roots and leaves of pea seedlings growing on a medium supplemented with lead and then during infestation by aphids. It should be noted that high levels of ABA in the presence of a sublethal dose of lead, both in the roots and the leaves, were recorded; these were markedly higher in the leaves than in the roots. Moreover, ABA accumulation also occurred as a result of *A. pisum* infestation, mainly at 24 h (+aphid variants).

On the other hand, it was demonstrated that heavy metals such as arsenic (As) led to a high expression of ABA biosynthesis genes such as *OsNCED2* and *OsNCED3* in rice (*Oryza sativa* L.), as well as causing the upregulation of four ABA signaling genes [36]. Moreover, as reported by Bücker-Neto et al. [37], an increase in the endogenous ABA levels in roots of *Typha latifolia* and *Phragmites australis* [38], in potato tubers [39] and also in rice plants was noted as a response to Cd treatment [40].

Plant–insect interactions in relation with the influence of other stress factors are generally poorly understood. In the published literature, there are no studies on the regulation of the expression of genes acting on phytohormone biosynthetic pathways by hormetic doses of heavy metals. It has been demonstrated that under heavy metal toxicity exposure, plants exhibit a rapid increase in ethylene production and reduced growth and development [41]. Furthermore, these authors have pointed out that most studies have focused on *ACS* or *ACO* gene expression levels, although other levels of regulation, such as post-transcriptional and post-translational modifications, can affect enzyme activities under heavy metal exposure. In turn, lead treatment yielded an increase in the transcript levels of *EIN2* in *Arabidopsis* seedlings, indicating a key role of this gene in heavy metal tolerance [42].

Our results indicate a significant induction of expression of the *ACS3* gene in the roots and leaves of pea seedlings growing under the influence of sublethal and hormetic doses of lead. Additionally, a strong upregulation of this gene under the influence of sublethal and hormetic doses of lead and during pea aphid infestation occurred. The *ACS3* gene encodes an essential enzyme in the biosynthesis of ET that catalyzes the synthesis of 1-aminocyclopropane-1-carboxylic acid (ACC), a precursor of ET from *S*-adenosyl methionine (AdoMet, SAM). Numerous studies have revealed that aphid feeding can stimulate ET synthesis upon different plant–aphid interactions [43]. The above authors demonstrated that ET production in Arabidopsis was significantly induced by aphid infestation. Furthermore, numerous studies have reported increased ethylene production in plants exposed to metals [44].

## 4. Materials and Methods

### 4.1. Plant Material and Growth Conditions

Seeds of pea (*Pisum sativum* L. cv. Cysterski) from the Plant Breeding Company in Tulce near Poznan in Poland were used during the experiments. Firstly, seeds were surface-sterilized using ethanol for 3 min and repeatedly rinsed with distilled water and finally left in an incubator for 6 h at 23 °C for imbibition. Next, the seeds were transferred to Petri dishes and immersed in a small amount of water for 66 h. The germinating seeds were transferred to dark hydroponic boxes with Hoagland medium. On the fifth day, the medium was replaced in all the hydroponic variants and lead was added to the medium at doses of 0.075 Pb(NO_3_)_2_ and 0.5 mM Pb(NO_3_)_2_ [6,14]. After the next four days, pea seedlings were infested with 20 adults of pea aphid. Samples for analyses were collected four days after Pb administration and prior to transferring the aphids onto the pea seedlings (at 0 h) and then after 24, 48 and 72 h of both stress treatments.

During the experiments, hydroponic cultures were aerated with an aeration system. All pea seedlings, including the control seedlings, Pb-treated seedlings and those growing in the presence of Pb and infested by *A. pisum*, were cultured in glass aquariums (30 cm × 22 cm × 28 cm) and protected with gauze. The experiment was conducted in a growth chamber at 22–23 °C and 65% relative humidity and with a light intensity of 130–150 μmol photons m^−2^s^−1^ with a 14/10 h (light/dark) photoperiod. The experimental variants were as follows: control pea seedlings cultured without Pb and not colonized by pea aphids (*Acyrthosiphon pisum*); pea seedlings growing in Hoagland medium with varied concentrations of Pb, i.e., 0.075 mM Pb (NO_3_)_2_ and 0.5 mM Pb(NO_3_)_2_; pea seedlings growing in Hoagland medium with varied concentrations of Pb and colonized by pea aphids *A. pisum* and pea seedlings growing in Hoagland medium colonized by pea aphids *A. pisum*. The objects of this study were the leaves and roots of pea seedlings.

### 4.2. Aphids and Infestation Experiments

*A. pisum* (Harris) aphids were reared on *P. sativum* L. cv. Cysterski in a growth chamber under conditions as specified above. On day 11 of culture, pea seedlings were infested with 20 wingless adult females of *A. pisum* using a fine paintbrush. The aphid populations were monitored throughout all the experiments [6,14]. The control pea seedlings were cultured with no addition of lead and not colonized by pea aphids.

### 4.3. Determination of Jasmonic Acid (JA) and Methyl Jasmonate (MeJA)

The analyses of jasmonic acid (JA) and methyl jasmonate (MeJA) were performed using GC–MS according to the method described by Fan et al. [45]. Leaves and roots of pea (1 g) were homogenized in liquid nitrogen and then with 200 ng of deuterium-labeled methyl jasmonate (d2-MeJA) and 200 ng of deuterium-labeled jasmonic acid (d5-JA) as internal standards in 20 mL of 80% (*v*/*v*) methanol. The suspension was left to stir overnight and then centrifuged at 10,000× *g* for 10 min. The residue was re-extracted with 80% methanol in two parts of 20 mL each. Combined supernatants were reduced to the aqueous phase by rotary evaporation, acidified to pH 2.0 with 7 M HCl and centrifuged at 10,000× *g* for 10 min to remove chlorophylls. The supernatant was partitioned three times against chloroform and then dried under vacuum. The residue was dissolved in 3 mL *n*-hexane and applied to a silica gel solid-phase extraction column (Backer-bound SPE silica gel, 500 mg, 3 mL; J.T.Backer, Philipsburg, NJ, USA). The column was washed with 5 mL *n*-hexane and then eluted with 6 mL of *n*-hexane:diethyl ether (2:1, *v*/*v*) with 0.5% (*v*/*v*) acetic acid. The eluate was evaporated and further purified by HPLC using a SUPELCOSIL ABZ + PLUS column (250 mm × 4.5 mm, 5 µm particle size; Supelco Inc., Bellefonte, PA, USA). The samples were dissolved in 200 µL of 20% methanol and chromatographed with a linear gradient of 20–80% methanol in 1% (*v*/*v*) formic acid in 20 min, with a flow rate of 1.0 mL/min at a temperature of 22 °C. The fractions collected at the retention times of 12.0–13.4 min for JA and 18.30–19.20 min for MeJA were evaporated to dryness, and in the case of JA, methylated with (Trimethylsilyl)diazomethane (Sigma-Aldrich, St. Louis, MO, USA), dissolved in 50 µL of methanol and analyzed by GC–MS-SIM (Auto-System XL coupled to a Turbo Mass, PerkinElmer, Waltham, MA, USA) using a DB-5 column (30 m × 0.25 mm, 0.5 µm phase thickness). The GC temperature program was 80 °C for 1 min, 80–160 °C at 10 °C/min and 160–230 °C at 5 °C/min, with a flow rate of 1 mL/min, injection port of 250 °C and electron potential of 70 eV. GC/MS-SIM analysis was performed by monitoring *m*/*z* 156, 161, 193, 198, 224, 226 and 229. The dwell time for all ions was 100 ms. The levels of JA and MeJA in the samples were determined from the ratio of peak area, calculated following d5-JA and d2-MeJA standards and expressed as nanograms per gram of fresh material (ng × g^−1^ FW).

### 4.4. Determination of Aminococyclopropane-1-carboxylic Acid (ACC)

Endogenous levels of aminococyclopropane-1-carboxylic acid (ACC) were determined using mass spectrometry combined with liquid chromatography (LC–MS). Leaves and roots of pea (300 mg) were homogenized in liquid nitrogen. For the extraction, 4 mL of 60% acetonitrile (*v/v*) in 2% HCOOH (*v/v*) was added, and a small amount of the antioxidant butylhydroxytoluol (BHT) was used. Additionally, 25 ng of 1-aminocyclopropane-2,2,3,3-d_4_-carboxylic acid as a standard was added. The mixture was shaken overnight, and then, 0.4 g of MgSO_4_ and 0.1 g NaCl were added and vortexed for 2 min. Next, the samples were centrifuged at 10,000× *g* for 10 min and the upper layer was collected. The pellet was extracted once again with 2 mL of acetonitrile and agitated for an additional 30 min and centrifuged. Combined supernatants were then dried in a stream of nitrogen, and the samples were reconstituted with 1 mL of 1M HCOOH and finally subjected to solid phase extraction using silica packed columns (Discovery^®^ DSC-18 SPE Tubes, Supelco Inc. Bellefonte, PA, USA). The columns were activated with 100% methanol and conditioned with 1 M HCOOH. Elution was performed using 80% methanol (*v/v*). For determination, the LCMS-8045 tandem mass spectrometer (Shimadzu Corp., Kyoto, Japan) was used. Chromatographic separation was carried out on a Kinetex^®^ 2.6 μm XB-C18 100 Å reverse phase column (150 × 2.1 mm, Phenomenex, Le Pecq, France). Water with 0.1% formic acid (*v*/*v*) (A) and methanol with 0.1% formic acid (*v/v*) (B) were used as the mobile phases. A linear gradient of 40–90% (*v/v*) of B for 7 min at a flow rate of 0.3 mL/min at 30 °C was used. In the mass spectrometry experiments, the samples were subjected to positive electrospray ionization (ESI), and ions were fragmented by collision-induced dissociation (CID). The ionization voltage was −3 kV. Analysis of the aminococyclopropane-1-carboxylic acid (ACC) content was based on the MRM transitions used for analyte quantitation (102 → 56 *m*/*z*) and for deuterated internal standard (106 → 60 *m*/*z*). Results were expressed as nanograms per gram of fresh material (ng × g^−1^ FW).

### 4.5. Extraction of Total RNA and Analysis by Reverse Transcription Polymerase Chain Reaction (RT-qPCR)

The extraction of the total RNA from roots and leaves was carried out using the miRNeasy Mini Kit (Qiagen, Venlo, the Netherlands), with the additional step of on-column digestion using an RNase-Free DNase Set (Qiagen, Venlo, The Netherlands). Evaluation of the quality and the quantity of the extracted total RNA was performed with a Nanodrop ND-1000 Spectrophotometer (Thermo Scientific Waltham, MA, USA), UV/VIS measurement and gel electrophoresis techniques. Synthesis of cDNA was performed with an NG dART RT Kit (EURx, Gdansk, Poland) using 0.5 µg of total RNA in a 20-µL reaction volume. The synthesized samples were then diluted with RNase-free water five times to a final volume of 100 µL before being used in the qPCR. Quantitative PCR was performed using the SensiFAST Probe No-ROX kit (Bioline Meridian Bioscience, Cincinnati, OH, USA) following the manufacturer’s protocol. Each 20-µL reaction contained the following: 5 µL cDNA template, 1 µL of 10 µM qPCR forward primer, 1 µL of 10 µM reverse primer, 10 µL of 2× SensiFAST Probe No-ROX Mix, 0.2 µL of 10 µM specific UPL probe (Roche, Basel, Switzerland) and 2.8 µL ddH_2_O. The qPCR was carried out on the LightCycler480 (Roche, Switzerland) under the following conditions: pre-incubation at 95 °C for 10 min followed by 45 cycles of 95 °C for 10 s, 60 °C for 30 s and 72 °C for 1 s. Each experiment, consisting of three biological replicates, was carried out in three technical replicates. Calculation of the relative expression levels was achieved using the 2^−∆∆Ct^ method, and the data were normalized to the CT values with the *PPA2* gene as a reference. The primers and UPL (Universal Probe Library) probes used in the qPCR were designed using ProbeFinder version 2.48 (Roche, Switzerland) and are listed in Table 4.

### 4.6. Statistical Analysis

All determinations were conducted within three independent experiments. The normality of the distribution of the ten traits, namely JA, MeJA, ACC, *LOX1*, *LOX2*, *OPR*, *JAR1*, *ACS3*, *NCED2* and *AO1*, was tested using the Shapiro–Wilk normality test [46] to verify whether the analysis of variance (ANOVA) met the assumption that the ANOVA model residuals followed a normal distribution. The homogeneity of the variance was tested using Bartlett’s test. Box’s M test tested multivariate normality and homogeneity of variance–covariance matrices. All the traits had a normal distribution. A two-way (time, variant) multivariate analysis of variance (MANOVA) was performed. Two-way analyses of variance (ANOVA) were carried out to determine the effects of time and variants as well as time × variant interaction on the variability of the observed traits. The relationships between the ten observed traits were estimated using Pearson’s linear correlation coefficients. Relationships within the observed traits are presented in heatmaps. The elementary comparisons between the specific levels of the analyzed factors were tested using the two-sample t-test for equal means for all the observed traits. To account for multiple testing, we used the Bonferroni correction. All analyses were performed independently for leaves and roots. The figures present the data obtained as means of triplicates for each variant along with standard errors of mean (SE). The GenStat v. 22 statistical software package (VSN International, Hemel Hempstead, UK) was used for the analyses.

## 5. Conclusions

In the present study, we revealed a significant induction of the expression of genes encoding enzymes for the biosynthesis of defense phytohormones such as JA/MeJA, ET and ABA under the influence of various concentrations of lead—i.e., at hormetic and sublethal doses. It should be emphasized that the sublethal dose of lead most strongly upregulated the tested genes, especially in the roots of *P. sativum* seedlings. A very strong induction of the selected genes was also noted in 72-h leaves and roots due to cross-talk of sublethal lead doses and *A. pisum* feeding. Additionally, our study has evidenced that feeding alone of the phytophagous insects with a piercing-sucking mouthpart enhanced the expression of these genes, both in the leaves and roots of pea seedlings. Moreover, the accumulation of defense phytohormones under stress was reported. The hormetic dose of lead strongly stimulated MeJA accumulation in the leaves of pea seedlings and upregulated some genes encoding enzymes for the biosynthesis of jasmonates, especially during the cross-talk of the hormetic lead dose and pea aphid infestation.

## Figures and Tables

**Table 4:** List of primers and UPL probes used for the qPCR.

Gene Symbol	Gene Name	Sequence Accession Number (NCBI GenBank or Pea RNA-Seq Gene Atlas)	EC Number	Forward Primer	Reverse Primer	UPL Probe Nr.
**Genes encoding jasmonate biosynthesis and signaling**						
*LOX1*	*Lipoxygenase 1*	PSU84198	1.13.11.12	tgacactgttcaaaaagacattga	gtgacccttttcgacagctt	31
*LOX2*	*Lipoxygenase 2*	PsCam035931	1.13.11.12	gccacaatggaagcaagtct	caagcctccaaagccaga	31
*JAR1*	*Jasmonic acid-amido synthetase JAR1*	PsCam027031	6.3.2.52	gaaaagggggtgtgatgcta	gcattcttgtatcccggattt	5
*OPR1*	*12-oxophytodienoate reductases*	AB104738	1.3.1.42	tggttaacgccttgaacaaat	gaggggtttcaactggatca	31
**Genes encoding ethylene biosynthesis**						
*ACS3*	*1-aminocyclopropane-1-carboxylate synthase 3*	AB049725	1.14.17.4	gagttaataatgttctgtttggctga	tgctggataataagggctaggt	9
**Genes encoding abscisic acid biosynthesis**						
*AO1*	*Aldehyde oxidase 1*	EF491598	1.2.3.1	gagttaataatgttctgtttggctga	taaacatacgctccgtgcag	9
*NCED2*	*9-cis-epoxycarotenoid dioxygenase*	AB080192	1.13.11.51	tcaaaatagagaaccaatcttctcc	tccatgtgtttgaaggtgatg	145
Reference gene						
*PP2A*	*Phosphoprotein phosphatase 2A*	Z25888	3.1.3.16	agctctgtgaagctgttggtc	cgaacatatgcaggaaccaat	31

## Data Availability

Data supporting reported results are deposited with the first author and corresponding author of the manuscript.
